# Cytarabine-based mobilisation yields higher CD34+ cell counts than cyclophosphamide regardless of prior daratumumab exposure in multiple myeloma

**DOI:** 10.3389/fonc.2026.1915658

**Published:** 2026-10-09

**Authors:** Wojciech Szlasa, Artur Borkowski, Monika Mordak-Domagała, Jacek Kwiatkowski, Katarzyna Malesa, Mateusz Sawicki, Jarosław Dybko

**Affiliations:** 1 Department of Haematology and Cellular Transplantation, Lower Silesian Oncology, Pulmonology and Hematology Center, Wrocław, Poland; 2 Department of Molecular and Cellular Biology, Faculty of Pharmacy, Wroclaw Medical University, Wrocław, Poland; 3 Department of Oncology and Hematology, Faculty of Medicine, Wrocław University of Science and Technology, Wrocław, Poland

**Keywords:** autologous transplantation, CD34+, cytarabine, daratumumab, multiple myeloma, stem cell mobilisation

## Abstract

**Introduction:**

Anti-CD38-based quadruplets such as daratumumab-bortezomib-lenalidomide-dexamethasone (D-VRd) or daratumumab-bortezomib-thalidomide-dexamethasone (D-VTd), where lenalidomide is not reimbursed, are the internationally recommended first-line treatment for transplant-eligible myeloma. There is ongoing debate about whether anti-CD38 exposure impairs stem cell collection. Most studies compare daratumumab-containing with non-daratumumab induction whilst keeping the mobilisation strategy constant. None have compared two mobilisation agents against each other in both daratumumab-exposed and daratumumab-naïve patients.

**Methods:**

We reviewed 66 patients (22 received Ara-C, 44 received CY) who were mobilised at a single centre in Poland between 2018 and 2024. No patients received plerixafor.

**Results:**

Patients who were mobilised with Ara-C collected more CD34+ cells than those who received CY, with a median of 12.6 (IQR 9.1–15.9) versus 7.7 (4.9–12.1) ×10⁶/kg (p=0.005), with comparable apheresis sessions. Previous D-VTd treatment did not reduce the overall yield (p=0.43). The benefit of Ara-C persisted in the D-VTd subgroup, despite heavy confounding between induction and mobilisation choice.

**Discussion:**

This is the first direct comparison of Ara-C and CY in the daratumumab era. Ara-C-based mobilisation was associated with higher CD34+ yields than CY in a plerixafor-free setting. The apparent advantage in D-VTd-treated patients is exploratory and requires prospective confirmation.

## Introduction

Autologous stem cell transplantation after high-dose melphalan is the main treatment for fit patients with newly diagnosed multiple myeloma (1, 2). The way the graft is collected is important. Both the total CD34+ yield and the side effects from mobilisation affect transplant outcomes (3). In most parts of the world, cyclophosphamide (CY) with G-CSF is the standard chemotherapy mobilisation method. However, Central Europe has a different practice. A Polish randomised trial showed that intermediate-dose cytarabine (Ara-C, 1600 mg/m²) plus G-CSF resulted in higher CD34+ yields with a reasonable safety profile (4). This builds on earlier research from the same group in lymphoid cancers (5), and the protocol has become a standard in several centres.

Current international guidelines, including the 2025 EHA-EMN recommendations, position anti-CD38-based quadruplets, specifically D-VRd or isatuximab-VRd, as the standard first-line induction for transplant-eligible myeloma (16). In Poland, lenalidomide is not included in the national drug reimbursement programme for induction therapy, so D-VTd is used as the locally available anti-CD38-containing quadruplet. The CASSIOPEIA trial showed that D-VTd was better than VTd in terms of response depth (6), but since CD38 is present on haematopoietic progenitors, there was concern that extended anti-CD38 treatment might reduce the stem cell pool. More recently, the PERSEUS trial evaluated daratumumab combined with bortezomib, lenalidomide, and dexamethasone (D-VRd) and confirmed that stem cell collection remained feasible after anti-CD38-containing induction and the overall yield was lower compared with VRd alone (15). Several other studies have tried to measure this effect. At Stanford, Uzun and colleagues found about a 9% decrease in CD34+ yield amongst 375 patients mobilised with G-CSF with or without plerixafor, with estimated mobilisation costs $23,000 higher in the exposed group (7). Two US centres combining data from 423 D-RVd-treated patients showed that early use of plerixafor could fully recover the shortfall, reaching a median yield of 9.0 ×10^6^/kg and a mobilisation failure rate of just 0.2% (8). A Greek study of 200 patients confirmed that collection after daratumumab was possible when plerixafor was available, although the daratumumab group required more plerixafor and had slower neutrophil recovery (11 days compared to 10 days) (9). A meta-analysis found a reduction of around 0.75 standard deviations in yield, even with optimised plerixafor, and noted delays in platelet recovery (10). Data from the MASTER and GRIFFIN trials showed median yields of 6.0 ×10^6^/kg after D-KRd and 8.3 ×10^6^/kg after D-RVd, with plerixafor used in 97% and 72% of patients, respectively (11).

These studies generally keep mobilisation consistent whilst varying the induction. The reverse question, whether the mobilisation agent can make up for any loss related to daratumumab, has not been widely studied. The DILEMMA study recently demonstrated that quadruplet regimen-treated patients can be effectively mobilised with chemotherapy (12). An Italian multicentre analysis looked at chemo-free mobilisation after D-VTd with promising results (13), but no study has directly compared two mobilisation agents in both daratumumab-exposed and unexposed patients.

Our centre provided a natural experiment to address this gap. Over the last six years, we adopted both D-VTd induction and Ara-C mobilisation around the same time, creating a group where all four combinations of a 2×2 matrix (induction × mobilisation) are represented. Another characteristic that distinguishes our data is the lack of plerixafor. This drug was not available during the study, which simplified interpretation compared to many large-centre studies. We aimed to find out if the type of mobilisation agent affects CD34+ yield, independently or in combination with daratumumab exposure.

## Materials and methods

### Study design and patients

We reviewed the records of every patient with multiple myeloma who had stem cell collection at our department (Lower Silesian Oncology Centre, Wrocław, Poland) from January 2018 to December 2024. The study adhered to the Declaration of Helsinki and received approval from the institutional review board. The database included 85 patients. Seven underwent allogeneic transplantation and were excluded. Among the remaining 78 autologous recipients, 68 had a documented chemo-mobilisation protocol on file (22 received Ara-C plus G-CSF, 46 received CY plus G-CSF). The other 10 lacked documentation. These were predominantly from the early study period (2018–2019), when mobilisation records were not systematically archived, and were not included in any analysis. CD34+ yield figures were available for 66 of the 68 patients (22 Ara-C, 44 CY), who form the primary analysis group.

### Treatment and mobilisation

Induction followed either the D-VTd schedule or a non-daratumumab backbone (VTd, VCD, or VD), selected by the treating physician based on drug availability and clinical judgement. Of the 66 evaluable patients, 27 had received D-VTd and 37 a non-daratumumab regimen. Two had incomplete induction records and were only included in the overall mobilisation comparison. The cycle number was guided by response assessment and scheduling.

For mobilisation, patients received either Ara-C 1600 mg/m² IV over three hours on day 1 or CY 2000 mg/m² IV on day 1, followed in both cases by G-CSF 10 µg/kg/day SC starting on day 5. The choice was made at the physician’s discretion. Ara-C adoption coincided with the introduction of D-VTd at our centre in 2023, so patients treated in more recent years were more likely to receive both. Apheresis began once peripheral CD34+ counts exceeded 20/µL, targeting ≥4.0 ×10^6^ CD34+ cells/kg (the minimum acceptable was 2.0 ×10^6^/kg for one transplant). Plerixafor was unavailable at our centre throughout the study and was not used for any patient.

### Endpoints and statistical analysis

The primary endpoint was the total CD34+ cell yield (×10^6^/kg). Secondary endpoints included the number of apheresis sessions and the proportion of patients who met the transplant threshold. Post-transplant events within the first 100 days, such as engraftment kinetics, infections, and neurotoxicity, were used as safety endpoints.

We present continuous data as medians with interquartile ranges (IQR) or means where appropriate. We used the Wilcoxon rank-sum test for comparisons between groups and Fisher’s exact test for proportions. Correlations were estimated using Spearman’s coefficient. The comparison of Ara-C versus CY yield was the pre-specified primary analysis. All other comparisons are exploratory, and we did not apply any multiplicity correction. The significance threshold was set at two-sided p<0.05. All calculations were performed in R 4.3.2 (R Foundation for Statistical Computing, Vienna).

## Results

### Patient characteristics

Of the 78 patients who received autologous transplants, 68 had documented chemo-mobilisation and 66 had data on cell yield. The groups receiving Ara-C and CY were fairly similar. The median age was 65 years (IQR 59–70) for Ara-C and 64 years (55–69) for CY. Males comprised 50% of the Ara-C group compared to 61% of the CY group. ISS stage III disease was found in 9% of evaluable patients in the Ara-C group and 17% in the CY group. One notable difference was that D-VTd induction was much more common amongst patients mobilised with Ara-C (73% vs 25%, p<0.001). This difference arose because our centre started using D-VTd and Ara-C at about the same time. Therefore, patients treated in more recent years were more likely to receive both treatments. **Table 1** summarises the baseline data.

**Table 1:** Patient characteristics by mobilisation regimen (evaluable population, n=66).

Variable	Ara-C (n=22)	CY (n=44)	p
Age, median (IQR), years	65 (59–70)	64 (55–69)	0.41
Male sex, n (%)	11 (50)	27 (61)	0.43
ISS stage III, n (%)*	2 (9)	8 (18)	0.48
D-VTd induction, n (%)	16 (73)	11 (25)	<0.001
Induction cycles, median (range)^†^	4 (2–6)	4 (2–4)	0.45
CD34+ yield, median (IQR), ×10^6^/kg	12.6 (9.1–15.9)	7.7 (4.9–12.1)	0.005
Apheresis sessions, mean	1.6	1.5	0.92
Melphalan 200 mg/m², n (%)	12 (55)	14 (32)	0.11

*ISS data available for 13/22 Ara-C and 39/44 CY patients. ^†^Cycle data available for D-VTd patients only (16 Ara-C, 11 CY).

### CD34+ yield by mobilisation agent

Patients mobilised with Ara-C collected significantly more CD34+ cells than those given CY. The median yield was 12.6 (IQR 9.1–15.9) compared to 7.7 (4.9–12.1) ×10^6^/kg (p=0.005, **Figure 1**). The two groups required a similar number of apheresis sessions, with a mean of 1.6 for Ara-C and 1.5 for CY (p=0.92). Every patient in both groups met the minimum threshold of 2.0 ×10^6^/kg.

**Figure 1:**
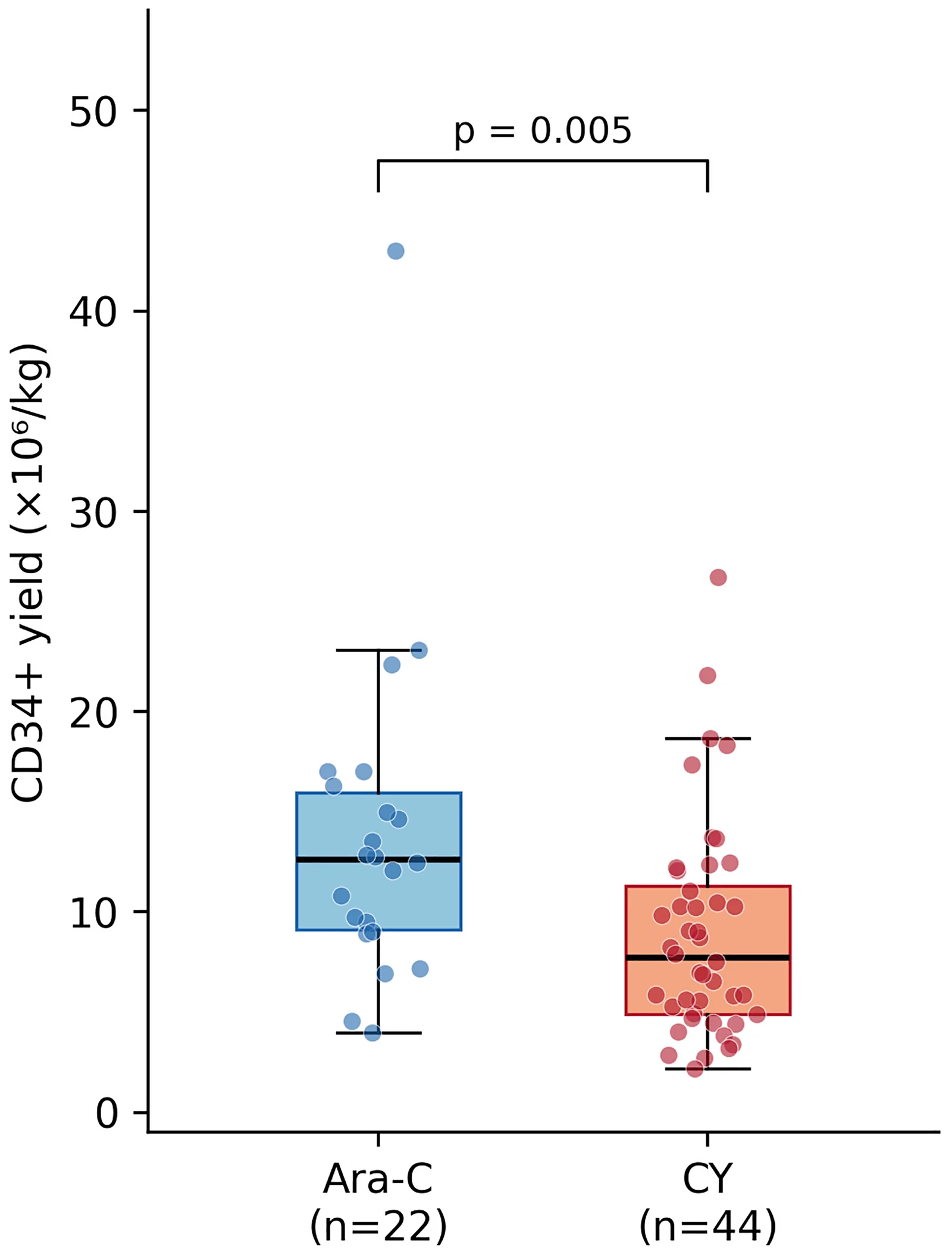
CD34+ cell yield by mobilisation regimen. Box-and-whisker plots with individual data points. Ara-C mobilisation produced a significantly higher median yield (12.6, IQR 9.1–15.9) than CY (7.7, IQR 4.9–12.1, p=0.005, Wilcoxon rank-sum test). n=22 (Ara-C), n=44 (CY).

### Effect of daratumumab exposure

When we compared yields based solely on the type of induction, D-VTd did not negatively affect collection. The median yield for D-VTd patients was 8.9 (IQR 5.9–12.8) compared to 9.8 (5.2–13.7) ×10^6^/kg for non-D-VTd patients (p=0.43, **Figure 2A**). Dividing the cohort into four subgroups by induction and mobilisation (**Figure 2B**) showed a consistent trend: Ara-C-mobilised patients had higher median yields in both induction strata (D-VTd subgroup: n=16 vs n=11, non-D-VTd subgroup: n=6 vs n=31). The subgroups were small, no formal interaction test was performed, and these findings should be viewed as descriptive and hypothesis-generating.

**Figure 2:**
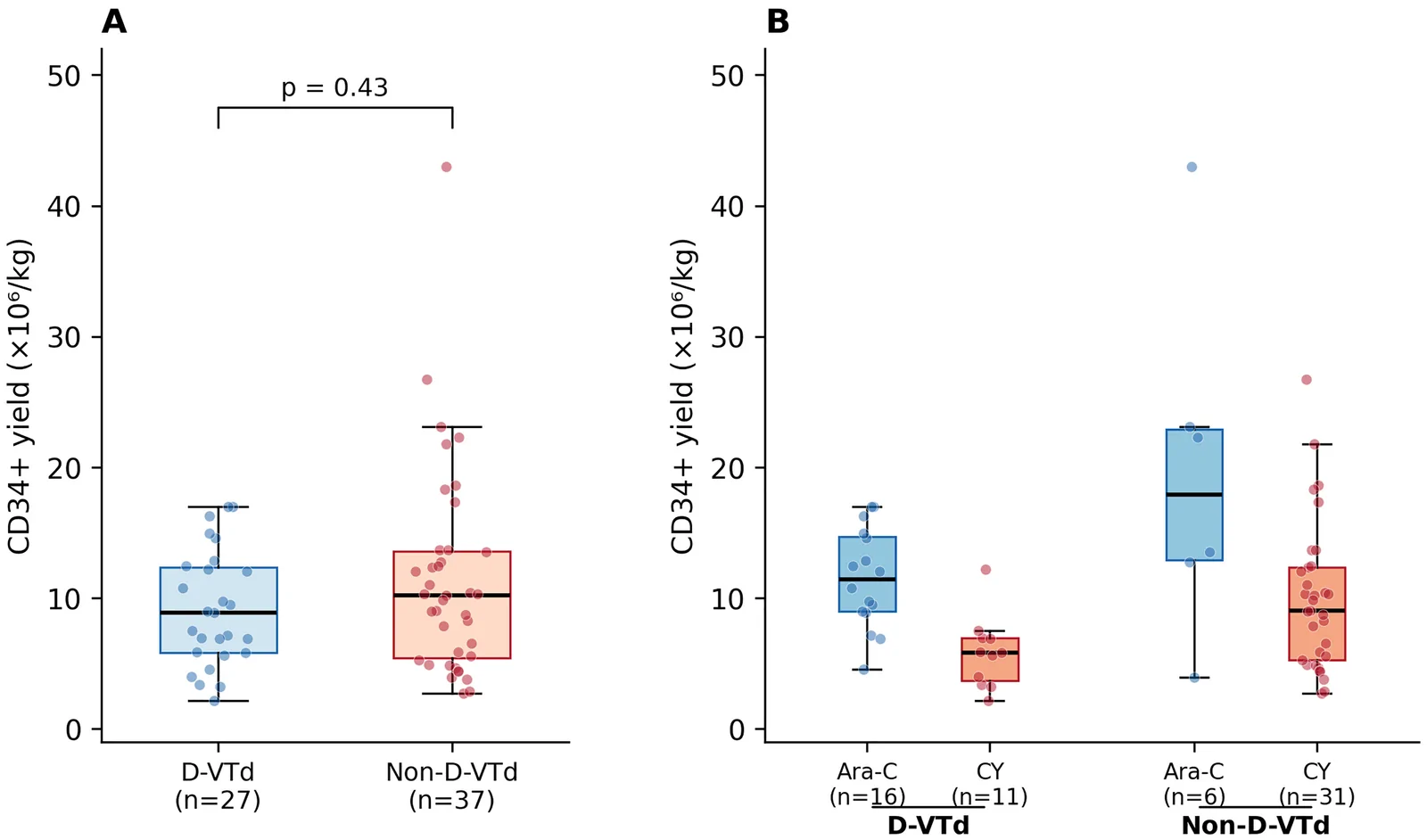
CD34+ yield by induction and mobilisation. **(A)** D-VTd (n=27) versus non-D-VTd (n=37) induction, no significant difference (p=0.43). **(B)** Exploratory four-group analysis. Ara-C-mobilised patients collected more in both induction strata. Subgroup sizes preclude formal interaction testing.

### Induction cycle number and CD34+ yield

For patients receiving D-VTd, the number of induction cycles ranged from 2 to 6 (**Figure 3**). A preliminary pooled analysis showed a positive Spearman correlation (ρ=0.42), but this was misleading. Most Ara-C patients had received 4 cycles, whilst CY patients were more evenly distributed across 2 to 4 cycles. When we examined each mobilisation arm separately, there was no significant link between the number of cycles and yield.

**Figure 3:**
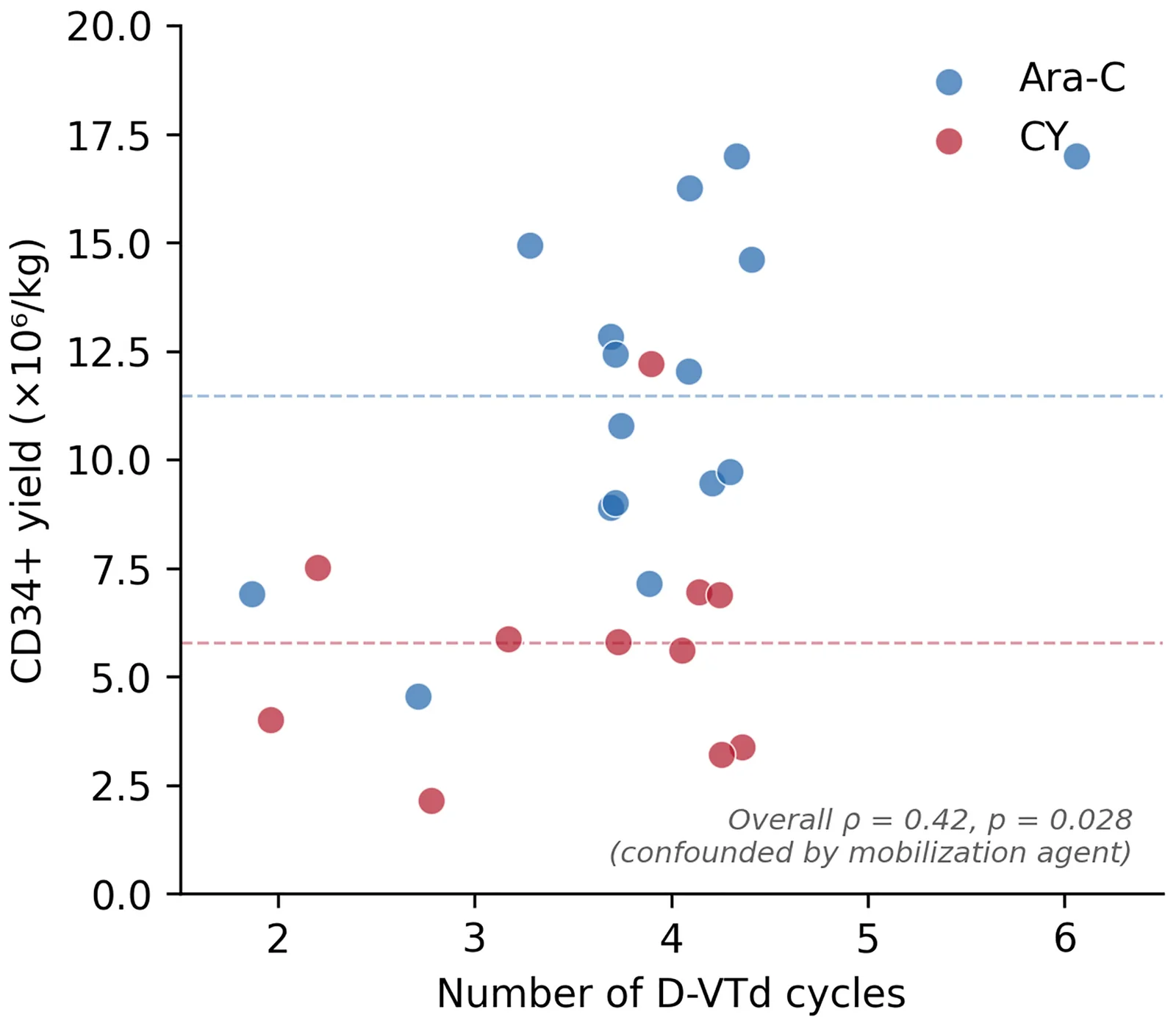
D-VTd cycle number versus CD34+ yield, colour-coded by mobilisation agent. The pooled positive correlation (ρ=0.42) is a confounding artefact: Ara-C patients (blue) out-collected CY patients (red) at every cycle count. Within-subgroup correlations were negligible.

### Correlation structure

A Spearman matrix covering the main clinical variables is shown. The only factor that significantly predicted CD34+ yield was Ara-C mobilisation (ρ=0.34, p=0.002). There was no correlation between D-VTd induction and yield (ρ=–0.15, p=0.19).

### Melphalan conditioning and post-transplant outcomes

The allocation of conditioning doses varied by era. Melphalan 200 mg/m² was used in 55% of Ara-C patients and 32% of CY patients (p=0.10, **Table 1**). The difference was not statistically significant. Complications within the first 100 days after transplant were similar between the induction groups, and every patient successfully engrafted (**Table 2**). The exact response category at the time of mobilisation was not consistently recorded in a form allowing a reliable distinction between CR and VGPR. However, the available clinical documentation confirmed that all patients had achieved at least VGPR before mobilisation, irrespective of the induction and mobilisation regimen.

**Table 2:** Post-transplant complications within 100 days by induction regimen.

Complication	D-VTd (n=27)	Non-D-VTd (n=37)	p
Infections, n (%)	3 (11)	3 (8)	0.69
Neurotoxicity ≥2, n (%)	1 (4)	3 (8)	0.63
Engraftment failure, n	0	0	—

Complication data were recorded for a subset of patients with complete post-transplant follow-up.

## Discussion

The main finding of this analysis is that Ara-C-based mobilisation produced significantly higher CD34+ yields than CY, and this advantage was not due to more apheresis sessions. This is the first study, to our knowledge, that compares these two mobilisation agents directly in a group including both daratumumab-exposed and daratumumab-naïve patients. D-VTd induction did not significantly affect overall stem cell collection. This result aligns with the prospective DILEMMA data (12) and with the experiences in Italy regarding chemo-free mobilisation after D-VTd (13).

To provide context for our yields, we compared them to the largest published series, although cross-study comparisons must be interpreted with caution given differences in patient populations, induction regimens, baseline disease burden, and apheresis protocols. Uzun et al. reported a median of 5.2 ×10^6^/kg in CD38-antibody-exposed patients and 5.5 ×10^6^/kg in unexposed patients using G-CSF alone. Mobilisation costs were $23,000 higher in the exposed group (7). Varga et al. achieved a median of 9.0 ×10^6^/kg in 423 DRVd-treated patients by using upfront plerixafor at two US centres. In their study, 94.3% of patients collected enough for two transplants (8). In the MASTER trial, even with 97% plerixafor usage, D-KRd-treated patients reached only 6.0 ×10^6^/kg. However, D-RVd recipients in GRIFFIN performed better at 8.3 ×10^6^/kg (11). Our Ara-C cohort achieved a median of 12.6 ×10^6^/kg without the use of plerixafor. The CY arm, with a median of 7.7 ×10^6^/kg, was numerically similar to the plerixafor-enhanced results from Varga et al. While these indirect comparisons suggest that chemo-mobilisation with Ara-C may reduce the need for plerixafor, they cannot establish equivalence or superiority across different patient populations.

The experience from Greece reported by Eleutherakis-Papaiakovou et al. offers a useful comparison, though again limited by differences in patient selection and mobilisation protocols. Among 200 patients (40 daratumumab-exposed and 160 not), 87.5% of the daratumumab group reached the target yield of ≥5 ×10^6^/kg, compared with 96.2% in the control group. The daratumumab group needed more plerixafor, more platelet transfusions, and had a longer delay in neutrophil recovery (11 vs. 10 days) (9). Our D-VTd + Ara-C subgroup achieved a mean of 11.5 ×10^6^/kg with a 100% success rate and no plerixafor. While this is encouraging, the subgroup comprised only 16 patients and differences in baseline characteristics preclude direct comparison with other cohorts. Duan et al.’s meta-analysis, which combined data from five studies, showed a daratumumab-related decrease in yield at SMD=–0.75 (95% CI –1.26 to –0.23) and noted delayed platelet engraftment (MD = 1.20 days) as another concern (10). Our patients showed no engraftment delay, though engraftment kinetics were not stratified by mobilisation agent and this observation should be interpreted cautiously.

What sets our dataset apart from existing literature is a mix of four features. First, the unique 2×2 structure: no other published cohort has enough patients in all four sections of the induction-times-mobilisation matrix to allow even preliminary cross-stratification. Second, the total lack of plerixafor. Most US centres commonly use plerixafor rescue (Chhabra et al. noted 97% usage in the MASTER D-KRd arm (11)), and its use is linked to both daratumumab exposure and low early CD34+ counts, complicating the results. Our cohort entirely avoids that variability. Third, our Ara-C protocol involves single-agent cytarabine at 1600 mg/m², following the protocol validated by Giebel et al. (5) and the randomised trial by Czerw et al. (4). Data from Poland by Drozd-Sokołowska et al. independently supports the biological benefit of this agent: in 58 patients needing repeat harvest after a previous transplant, Ara-C had an 84% success rate, compared to 53% for CY (14). Fourth, we actively sought out confounding factors instead of ignoring them. The cycle-number analysis (**Figure 3**) illustrates this point: the combined ρ of 0.42 vanished once we factored in the mobilisation agent. This highlights how easily selection bias can disguise itself as a dose-response relationship in observational transplant data.

We need to be clear about the main threat to internal validity. Mobilisation agent and induction were closely linked: 73% of Ara-C patients received D-VTd compared to 25% of CY patients (p<0.001). Both treatments were introduced in the same time period, so the relationship is temporal rather than causal. If D-VTd really reduces yield, this confounding factor harms Ara-C. This suggests the true benefit could be greater than what we observed. Alternatively, if some unmeasured patient factor influenced both choices, it could lead to selection bias. We looked into using propensity-score methods, but the sample was too small. Preliminary multivariable models showed negative R² values, which clearly indicates overfitting due to too many covariates relative to events.

The clinical relevance of obtaining very high CD34+ cell yields should be interpreted cautiously. Tandem autologous transplantation is not routinely required for all patients, although it may still be considered in selected patients with genetically defined high-risk disease (16). Surplus cryopreserved cells may also preserve the option of a later autologous transplant. In addition, previously collected autologous CD34+ cells can potentially be used as a stem cell boost in patients who develop prolonged haematotoxicit after subsequent BCMA-directed CAR T-cell therapy (17). This possible future use is not an indication for targeted overcollection or for a more intensive mobilisation procedure. It simply provides another potential use for cells obtained during an otherwise indicated mobilisation. Cytarabine is not an established antimyeloma treatment, and our study did not assess changes in disease response during mobilisation. We therefore interpret the advantage of Ara-C as greater mobilisation efficacy rather than as evidence of tumour cytoreduction. Mobilisation-related toxicity was not systematically recorded in our retrospective database. Data on cytopenia duration, febrile neutropenia, infections, transfusion requirements, intravenous antibiotic use and hospitalisation were incomplete and could not be compared reliably between the Ara-C and CY groups. We did not attempt to reconstruct these outcomes from selected medical records because differences in documentation over the study period would introduce a substantial risk of ascertainment bias. Although all patients ultimately engrafted, detailed neutrophil and platelet recovery kinetics and post-transplant transfusion requirements were not available according to mobilisation regimen. Consequently, the present study compares mobilisation efficacy but does not allow a comparative assessment of safety or the overall risk–benefit balance of Ara-C versus CY. This substantially limits the practical interpretation of our findings and should be addressed in a prospective study with predefined toxicity and engraftment endpoints.

Although avoidance of plerixafor reduces drug-acquisition costs, it does not necessarily reduce the total cost of mobilisation. Comparative economic studies of outpatient G-CSF with planned or risk-adapted plerixafor versus cyclophosphamide-based chemomobilisation have yielded heterogeneous results. In some studies, the higher cost of plerixafor was offset by reduced hospitalisation, toxicity and resource utilisation, whereas other analyses found plerixafor-based mobilisation to remain more expensive (18–23). These findings cannot be directly extrapolated to intermediate-dose cytarabine, for which no direct economic comparison with G-CSF with plerixafor is available. Studies of cytarabine-based mobilisation have primarily evaluated efficacy and toxicity rather than total costs (4, 24). As mobilisation-related hospitalisation, transfusion requirements, antibiotic use and other cost components were not captured in our study, no pharmacoeconomic conclusions can be drawn from the absence of plerixafor use in our cohort.

Our findings should be interpreted in the context of the evolving treatment landscape. The majority of published mobilisation data after anti-CD38 therapy come from centres using D-VRd, which includes lenalidomide, a drug with known myelosuppressive effects that may independently impair stem cell mobilisation. Our cohort used D-VTd (thalidomide-based), which may differ in its impact on the haematopoietic stem cell compartment. Whether our results are generalisable to D-VRd-treated patients requires dedicated study.

We demonstrate a difference between Ara-C and CY in this cohort. We do not demonstrate that Ara-C is superior specifically after daratumumab. The latter remains exploratory.

The typical limitations of retrospective, single-centre studies apply here. Mobilisation was not randomised. We cannot resolve the Ara-C/D-VTd confounding factor without a larger, more balanced dataset or a prospective design. The non-D-VTd plus Ara-C subgroup had just 6 patients, which is too small to draw any meaningful conclusions about the effect of Ara-C in the non-daratumumab setting. Findings in this subgroup are purely hypothesis-generating. ISS staging was absent in about one fifth of cases (21%). Since plerixafor was never available, our results may not directly apply to settings where it is a standard rescue option. We did not capture mobilisation-related toxicity data such as febrile neutropenia, hospitalisations, transfusion requirements, intravenous antibiotic use, or duration of cytopenia, which precludes a safety comparison between Ara-C and CY mobilisation. Although all patients had achieved at least VGPR before mobilisation, the exact response category at that specific time point was not systematically recorded. We were therefore unable to compare CR versus VGPR between the groups or include response depth as a covariate. Residual confounding related to differences in response depth cannot be excluded, although none of the groups included patients with only PR, progressive disease or inadequate disease control.

In conclusion, Ara-C-based mobilisation was associated with higher overall CD34+ yield than cyclophosphamide in this plerixafor-free cohort. The apparent pattern favouring Ara-C in D-VTd-treated patients is exploratory and requires prospective confirmation with balanced groups and formal interaction testing.

## Data Availability

The original contributions presented in the study are included in the article/supplementary material. Further inquiries can be directed to the corresponding author.
